# Four-step eco-friendly energy efficient recycling of contaminated Nd_2_Fe_14_B sludge and coercivity enhancement by reducing oxygen content

**DOI:** 10.1038/s41598-021-01382-4

**Published:** 2021-11-15

**Authors:** Syed Kamran Haider, Dongsoo Kim, Young Soo Kang

**Affiliations:** 1 Convergence Research Center for Development of Mineral Resources, Korea Institute of Geoscience and Mineral Resources, 124, Gwahakro, Yuseonggu, Daejeon, 34132 Korea; 2 Powder and Ceramics Division, Korea Institute of Materials Science, 797, Changwondaero, Seongsangu, Changwon, Gyeongnam 51508 Korea; 3 Department of Chemistry, Sogang University, 35, Baekbeomro, Mapogu, Seoul, 04107 Korea

**Keywords:** Magnetic materials, Magnetic properties and materials, Sustainability, Chemistry, Materials science

## Abstract

Complete recycling of Nd_2_Fe_14_B sludge by chemical methods has gained significance in recent years, however, it is not easy to recycle highly contaminant sludge and obtain product with good magnetic properties. Herein we report a simple four-step process to recycle the Nd_2_Fe_14_B sludge containing ~ 10% of contaminants. Sludge was leached in H_2_SO_4_ and selectively co-precipitated in two steps. In the first co-precipitation, Al^3+^ and Cu^2+^ were removed at pH 6. Thereafter, in the second co-precipitation Fe^2+^ and RE^3+^ sulfates were converted to the Fe and RE hydroxides. By annealing at 800 °C RE and Fe hydroxides precipitates were converted to the oxides and residual carbon was oxidized to CO_2_. After the addition of boric acid, Fe and RE oxides were reduced and diffused to the (Nd-RE)_2_Fe_14_B by calciothermic reduction diffusion. Removal of CaO by washing with D.I. water in glove box reduced the oxygen content (~ 0.7%), improved crystallinity and enhanced the magnetic properties significantly. Coercivity increased more than three times (from 242.71 to 800.55 kA/m) and M_r_ value was also enhanced up to more than 20% (from 0.481 to 0.605 T). In this green process Na_2_SO_4_ and Ca(OH)_2_ were produced as by-product those are non-hazardous and were removed conveniently.

## Introduction

Among permanent magnets, Nd_2_Fe_14_B type hard magnets exhibit the highest recorded BH_max_^1–5^. They have drawn attention due to their applications in modern appliances which lead to large market demand and a rapid increase in their production^6–11^. A huge amount (21%) of rare earth elements (RE) are being consumed for the synthesis of permanent magnets. RE resources are depleting and the cost of RE extraction from the ores is continuously soaring, hence recycling of the Nd_2_Fe_14_B sludge is becoming an important area of modern research.

A large quantity (~ 30%) of the Nd_2_Fe_14_B sludge is produced in the cutting and grinding process and more than 95 wt% of it, is recyclable^12^. However, because of costly physical recycling processes and high level of contamination, usually, recycling magnet sludge is not economically feasible in most part of the world. Nd_2_Fe_14_B sludge mainly consists of oxidized particles of Nd_2_Fe_14_B with different RE, C, Al, and d-block transition metals (e.g. Cu, Co, Zn, Mn, Cr, Ni). Al, Zn, Mn, Cr, and Ni come from the protective coatings, those are applied to avoid the corrosion of bulk magnet surface. Cu is added to the sintered Nd_2_Fe_14_B magnets to decouple the magnetic grains to stop the fast flip-over of the magnetic domains and enhance the coercivity^11^. Co addition to enhances the M_r_ value and curie temperature. Sludge can have a high quantity of carbon because of the mixing of machine oil and lubricant during the cutting process. All these contaminants reduce the value of sludge.

Commonly used physical method for recycling of Nd_2_Fe_14_B sludge is a multi-step process which requires a huge amount of chemicals, energy, and produces hazardous wastes as byproduct^13^. These wastes include oxides (of carbon, sulfur, and nitrogen), dangerous metals (e.g. As), organic solvents, RE vapors and electrolytes. Several chemical methods have been introduced for the recovery of RE from Nd_2_Fe_14_B magnets scrap/sludge^12–26^ but complete recycling of Nd_2_Fe_14_B, via chemical route is relatively new field. Haider et al.^12^ and Yin et al.^13^ have recently introduced chemical methods. These methods are very innovative and useful, but they deal with sludge with a low level of contamination. It is very difficult to control the contamination in the sludge, hence it was required to introduce a new method to recycle this kind of sludge with good magnetic properties.

**Figure 1:**
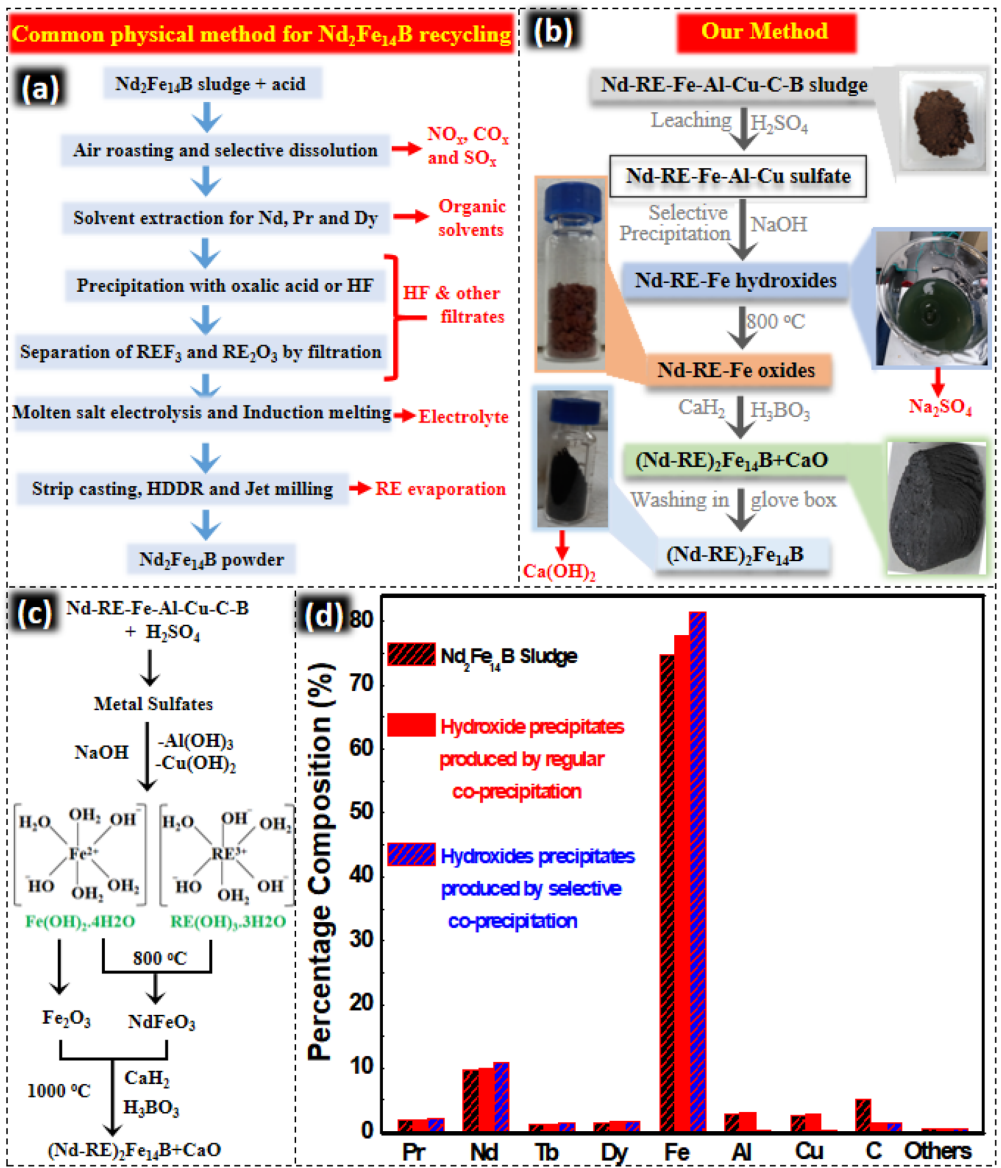
(**a**) Common physico-chemical method for the recycling of Nd_2_Fe_14_B sludge used these days^12^. (**b**) Our experimental process. Waste products produced in both methods are shown in red color. (**c**) Chemistry of the process. (**d**) Composition of the sludge and hydroxides precipitates produced after regular and selective co-precipitation.

Herein we propose a time and energy efficient-recycling method for recycling of Nd_2_Fe_14_B sludge which is equally useful for sludge with high contamination and variable composition. Na_2_SO_4_ and Ca(OH)_2_ are produced as byproducts, those are non-hazardous and easy to remove. Magnetic properties are further enhanced by the removal of contamination and reduction of oxygen content. A comparison of between our method and the common physico-chemical^12^ method used for the recycling of Nd_2_Fe_14_B sludge is given in the Fig. 1a, b.

## Experimental section

### Materials

All chemicals used in this work, including neodymium (III) chloride hexahydrate (Nd_2_(SO_4_)_3_·6H_2_O), sodium hydroxide (NaOH), boric acid (H_3_BO_3_), calcium hydride (CaH_2_), sulfuric acid (H_2_SO_4_), ethyl alcohol (C_2_H_5_OH) and acetone (CH_3_COCH_3_) were analytical grade and obtained from Sigma-Aldrich Co. Magnet sludge was obtained during the cutting process of Nd_2_Fe_14_B.

### Experimental process

Magnet sludge obtained during the cutting of Nd_2_Fe_14_B magnet was dissolved in 2M H_2_SO_4_ by leaching process. ICP analysis (by inductively coupled plasma atomic emission spectrometer) of the leachate showed that it consisted of Nd, Pr, Tb, Fe, Cu, Al, and C (Fig. 1a). Total RE:Fe molar ratio in the leachate was calculated as 15.8:83.8. In order to compensate for the lower RE concentration in the leachate and to make up the RE:Fe molar ratio as 15:77, extra Nd_2_(SO_4_)_3_·6H_2_O was added to the leachate solution.

Co-precipitation of leachate was performed with a 3.5M NaOH solution which was added drop-by-drop, to raise the pH of the leachate solution up to 6. At pH 6, Al, Zn, and Cu chlorides were converted to their respective hydroxide precipitates. These precipitates were removed from the reaction mixture by centrifugation. The left-over reaction mixture was co-precipitated by further addition of NaOH solution. By maintaining the pH at 13, the solution was stirred for 30 min, which lead to the formation of RE and Fe hydroxides precipitates. The precipitates were washed thrice with de-ionized water to remove the Na_2_SO_4_ and NaOH and then annealed at 800 °C to convert all Fe and RE hydroxides to oxides. The annealing was done for 30 min while the air was flowing in the furnace. Produced oxide particles were mixed with boric acid and CaH_2_ in glove box and the mixture was pressed to the pellet. Pellet increases the physical contact between the constituents e.g. calcium hydride, boric acid, oxides of RE and Fe, which is useful for the efficient R-D (reduction-diffusion). Boric acid was added in such a way that the molar ratio of RE:Fe:B was kept as 15:77:8. Oxides (+ boric acid):CaH_2_ weight ratio was fixed as 1:1.

RE and Fe oxides (mixed with CaH_2_) were reduced and diffused by annealing at 1000 °C for 3 h. Products obtained after R-D were washed with water in the glove box repeatedly to remove CaO and rinsed with acetone twice. Finally, vacuum drying was done and recycled (Nd-RE)_2_Fe_14_B powder was stored in inert conditions. Flow diagram of experimental process and summary of chemical reactions during the process are provided as Fig. 1b, c. Advantages (energy efficient and cost effective) of the chemical method over the commonly used method are described in the Figs. S1 and S2, in the supporting information.

### Characterization

The concentration of the elements in the leachate was determined by the inductively coupled plasma atomic emission spectrometer (ICP-AES, Shimadzu). Crystal structure and phases were determined by X-ray diffraction (XRD) patterns using a Rigaku Diffractometer (XRD, Rigaku). The morphology, size, and elemental distribution were observed with field emission scanning electron microscope (FE-SEM, Merlin), conventional transmission electron microscopy (TEM, JEM-2100F), and aberration-corrected TEM (ARM-200F) with energy- dispersive X-ray spectroscopy (EDS). TEM was operated at the accelerating voltage of 200 kV. Magnetic properties (M-H curves) of final product were measured by Physical Property Measurement System (PPMS, Evercool II–9T) in the vibrating sample magnetometer mode. LECO ON-736 analyzer was used to determine the oxygen content in (Nd-RE)_2_Fe_14_B.

Specimens for TEM were prepared by focused ion beam (FIB- NX2000, Hitachi) using the lift-out technique. For TEM measurement, the sample was treated as the same process reported by Kim et al.^27^ and orientation of the sample along the required zone axis was confirmed by using electron backscatter diffraction (EBSD) by TEAM™ Pegasus, Ametek Co. Ltd. USA.

## Results and discussion

Magnet sludge produced during the cutting of Nd_2_Fe_14_B was dissolved in the H_2_SO_4_. Overall composition of the sludge before and after the precipitation is provided in Fig. 1d. Sludge mainly consisted of RE (Nd, Pr, Tb, Dy) Fe, Cu, Al, and C. Traces of other elements (e.g. Ho, Zr, Ga, Ni, Co) were also detected in the ICP analysis but their total concentration was ~ 0.4%. Effect of these small impurities on the magnetic properties of the final product was studied and provided in the supporting information. Cu, Al and C were ~ 10% those could significantly reduce the magnetic properties of the (Nd-RE)_2_Fe_14_B produced from them. After leaching of sludge in the H_2_SO_4_, next step was the selective precipitation to remove Al^3+^ and Cu^2+^. Precipitation of multivalent (e.g. Al^3+^ and Cu^2+^) ions from the solution depends on many factors e.g. oxidation state, Ksp value, the concentration of other ions, precipitating agent, and temperature. Precipitation of Fe^3+^, Al^3+^, and Cu^2+^ from their chloride solution occurred at pH values of 3.5, 5.0, and 6.0^28^. However, different results were observed, when the solution of acid mine drainage containing Fe^3+^, Al^3+^, and Cu^2+^ was co-precipitated^28^. In the acid mine drainage experiment, Fe^3+^, Al^3+^, and Cu^2+^ chlorides were precipitated at pH 3.5, 4.5, and 5.5, respectively^28^. Precipitation of Al^3+^ and Cu^2+^ at pH value of 5.5 was also observed^29^. Different pH of the precipitation for Al^3+^ and Cu^2+^ in the previous studies indicated is a complicated process. It is commonly observed that Fe^3+^ precipitates completely at pH value ~ 3, however, Fe^2+^ precipitates at pH value of ~ 7^30^. In our study, when pH approached 4, Al (Ksp constant = 1.9 × 10^−33^) started to precipitate.

Ksp constants of Cu(OH)_2_ is 1.6 × 10^−19^ and it was next to be precipitated hence precipitated out between the pH values of 5–6. Almost ~ 90% hydroxides of Al and Cu were separated in the form of precipitates at pH 6, they were removed from the leachate solution by centrifugation. Separated Al and Cu hydroxides were analyzed, and analysis details are provided in the supporting information (Figs. S3, S4).

Fe in the leachate exists as Fe^2+^ which does not precipitate below pH 6 but slightly (~ 2%) precipitates at pH 6 (Fig. S5). However, when pH value exceeded 6, Fe^2+^ (Ksp constant = 7.9 × 10^−15^) started to precipitate as Fe(OH)_2_. Soe et al.^31^ reported that Nd^3+^, Pr^3+^, Dy^3+^, and Tb^3+^ also start to precipitate at pH value of ~ 7 and similar was observed in our study.

Co-precipitation was stopped at pH values of 10, 11, 12 and 13 in four different experiments. The maximum percentage yield was obtained at pH value of 13 (Fig. S6). At pH 13, a mixture of hydroxides of RE, and Fe, was obtained with traces of some impurities e.g. Cu, Al, Ho, Zr, Ga, Ni, Co (less than 1%). In a separate experiment, selective precipitation was not performed and all the elements in the leachate solution were co-precipitated, hence, a mixture of RE, Fe, Al, and Cu hydroxide was obtained.

**Figure 2:**
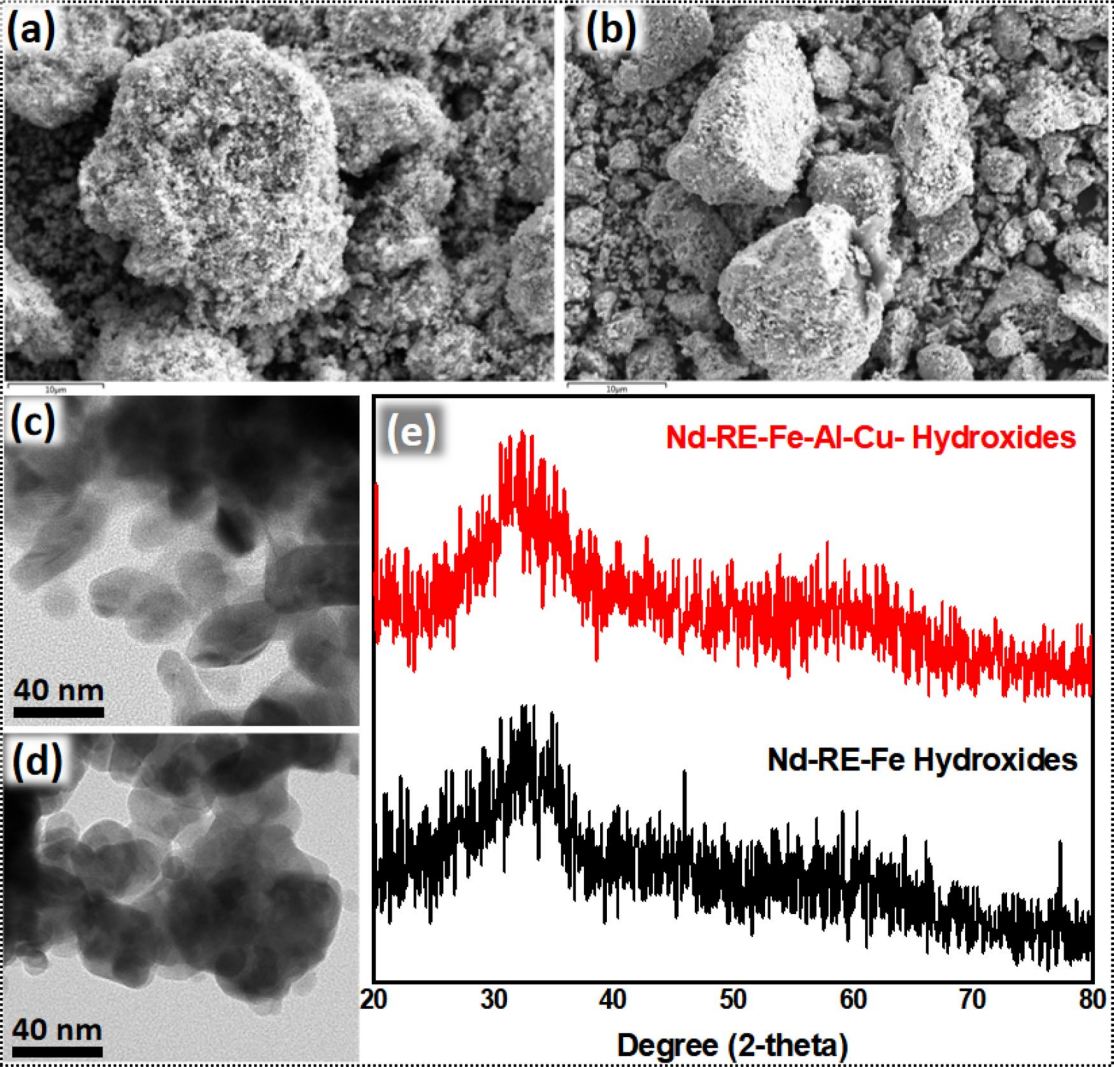
SEM image of hydroxide precipitates of (**a**) Nd-RE-Fe-Al-Cu and (**b**) Nd-RE-Fe. TEM image of hydroxide precipitates of (**c**) Nd-RE-Fe-Al-Cu and (**d**) Nd-RE-Fe. (**e**) XRD patterns of Nd-RE-Fe-Al-Cu and Nd-RE-Fe hydroxide precipitates.

Hydroxides obtained by co-precipitation were aqua complexes of RE and other metals. Being amorphous, these hydroxides could not be detected by XRD (Fig. 2e) analysis. SEM image of the hydroxide precipitates produced by regular and selective co-precipitation (Fig. 2a, b) confirmed that hydroxide particles were of irregular morphology and size (Fig. 2c, d). TEM analysis revealed that the average size of the hydroxide precipitates was ~ 25 nm. TEM-EDS images (Figs. S7, S8) confirmed that oxides of all the metals are homogeneously mixed.

**Figure 3:**
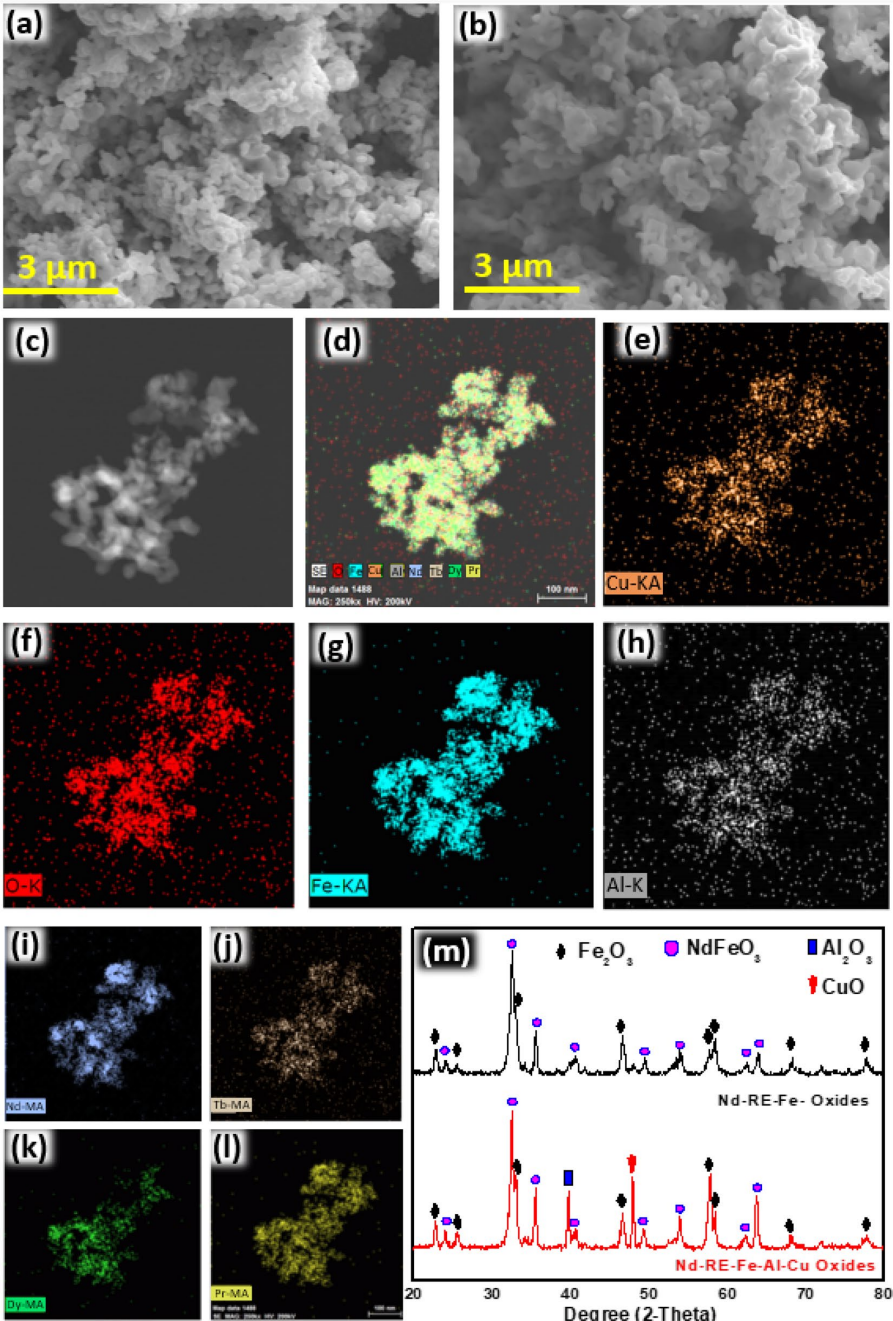
SEM images of (**a**) Nd-RE-Fe-oxide (**b**) Nd-RE-Fe-Al-Cu-oxide (**c**–**l**) TEM and TEM-EDS images of Nd-RE-Fe-Al-Cu-oxide (**m**) XRD patterns of Nd-RE-Fe-oxide and Nd-RE-Fe-Al-Cu-oxide.

Most of the carbon was removed during the precipitation and centrifugation but still, noticeable quantity was detected in the hydroxide precipitates (Fig. 1d). Leftover carbon was removed by the annealing in air at 800 °C. Oxidation at 800 °C converted all the C to CO_2_. Meanwhile, annealing also converted hydroxide precipitates to the oxides. SEM analysis revealed that the average particle size of the oxide particles was ~ 150 nm (Fig. 3a, b).

**Figure 4:**
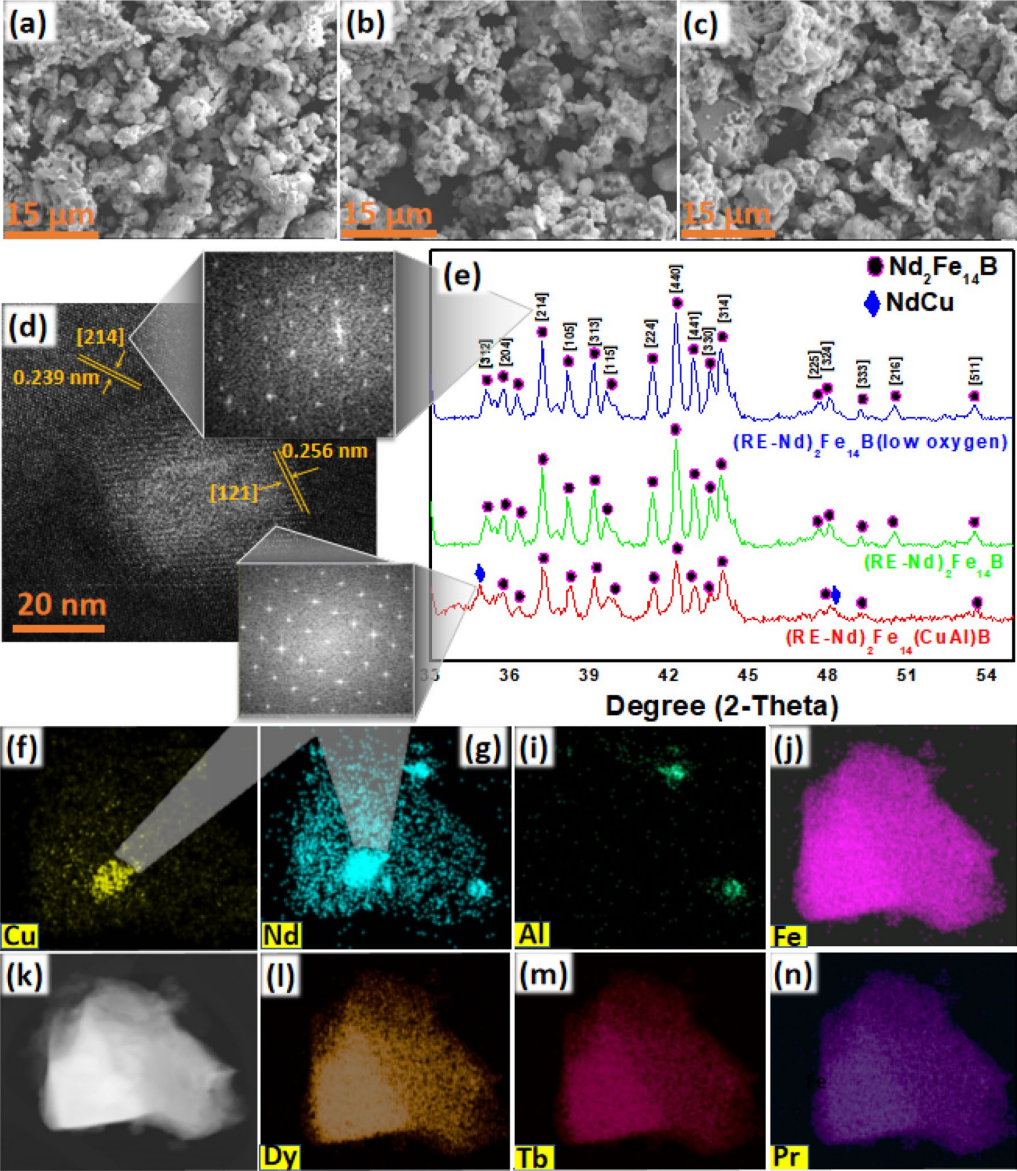
SEM images of (**a**) (Nd-RE)_2_Fe_14_(AlCu)B, (**b**) (Nd-RE)_2_Fe_14_B, (**c**) (Nd-RE)_2_Fe_14_B-(low oxygen), (**d**) HRTEM and SEAD patterns of (Nd-RE)_2_Fe_14_(AlCu)B, (**e**) XRD patterns for all three products, (**f**–**n**) TEM-EDS images of (Nd-RE)_2_Fe_14_(AlCu)B.

XRD confirmed the presence of Fe_2_O_3_ and REFeO_3_ phases in the oxide mixture (Fig. 3m). The mechanism of formation of these oxides is given in Fig. 1c. Peaks of Cu and Al oxides were also observed in the XRD patterns of oxides produced from Nd, RE, Fe, Al, Cu hydroxide precipitates. TEM-EDS images reveal that all RE, Fe, Cu, and Al are distributed evenly throughout the oxide intermediates (Fig. 3c–l). Co-precipitation brought the Fe_2_O_3_ and REFeO_3_ particles very close. This homogeneous distribution is very effective for the efficient reduction diffusion process. Oxides produced from both the selective and regular co-precipitation were mixed with the boric acid and CaH_2_ in two separate experiments. These mixtures were reduced and diffused at 1000 °C to obtain (Nd-RE)_2_Fe_14_(AlCu)B and (Nd-RE)_2_Fe_14_B. Both of these products contained CaO byproduct, and were washed with water to remove it. (Nd-RE)_2_Fe_14_(AlCu)B was washed in the air while (Nd-RE)_2_Fe_14_B was divided into two parts. One part was washed in the air and other part was washed in the glove box to minimize the exposure to oxygen. In this way three different products (Nd-RE)_2_Fe_14_(AlCu)B, (Nd-RE)_2_Fe_14_B and (Nd-RE)_2_Fe_14_B (low oxygen), were obtained. To determine the detailed structure of the products XRD, SEM, SEM–EDS, TEM, TEM-EDS, and HRTEM analysis were performed. Nd_2_Fe_14_B, Dy_2_Fe_14_B, Tb_2_Fe_14_B, and Pr_2_Fe_14_B have similar crystal structures, hence their XRD patterns are also very similar. It was very hard to distinguish between all four RE_2_Fe_14_B phases with the help of XRD. Hence all these phases are marked as Nd_2_Fe_14_B (JCPSD #36-1296) in the XRD patterns shown in Fig. 4e. NdCu (JCPSD #337-1037) phase was also detected in the XRD analysis of (Nd-RE)_2_Fe_14_(AlCu)B. To evaluate the crystallinity of the of (Nd-RE)_2_Fe_14_(AlCu)B, SAED patterns (Fig. 3b) of the Nd_2_Fe_14_B and ZnCu were obtained. With d-spacing value of 0.239 nm, [214] facet of (Nd-RE)_2_Fe_14_B was detected. Any peak of Al or Al alloy was not detected in the XRD, however, in TEM-EDS image, Al was detectable (Fig. 4i). This may refer to that Al or Al alloy was not crystallized well during reduction-diffusion or maybe oxidized during the washing with water and became amorphous. However, [121] facet of NdCu was identified in the HRTEM with the d-spacing value of 0.256 nm (Fig. 4d).

SEM images in Fig. 4a–c revealed that the magnetic particles had irregular morphology and the size varied from 0.3 to 10 μm. It was determined that the average particle size of all three particles was ~ 1.8 µm (Fig. 4a–c)*.* Different studies have revealed that Dy, Pr Tb, and other heavy RE are substituted inside the Nd_2_Fe_14_B crystal lattice^32–43^. In our study HRTEM and SEM–EDS images (Fig. 4l–n) also confirmed it. HRTEM shows that the crystal lattice d-spacing at [214] facet is 0.239 nm, which is slightly smaller (241 nm) than NdFe_14_B [214] facet. Slight reduction in the d-spacing may also indicate the substitution of Pr, Tb, or Dy in the crystal lattice. TEM-EDS images of (Nd-RE)_2_Fe_14_(AlCu)B confirmed the homogeneous mixing of all RE, indicate the substitution of Dy, Pr, and Tb in the Nd_2_Fe_14_B crystal lattice (Fig. 4f–n). TEM and TEM-EDS images of (Nd-RE)_2_Fe_14_B and (Nd-RE)_2_Fe_14_B (low oxygen) are provided in supporting information as Figs. S9, S10 and S11.

Oxygen content in the commercial (Nd-RE)_2_Fe_14_B powders is ~ 0.4%. This one of the reasons that commercial (Nd-RE)_2_Fe_14_B powders exhibit excellent magnetic properties. In the rare earth based magnetic particles produced by the R-D, washing with the water is employed to remove the CaO, byproduct of the R-D process^44^. On average, (Nd-RE)_2_Fe_14_B magnetic particles produced by the reduction diffusion process contains ~ 2% of oxygen. It was concluded that oxygen content reduces the crystallinity Nd_2_Fe_14_B because no oxide is detected in the XRD patterns. To solve the oxidation problem, (Nd-RE)_2_Fe_14_B was washed in the glove box. Before washing the (Nd-RE)_2_Fe_14_B with the water in the glove box, nitrogen gas was blown in the water, which further removed the dissolved oxygen in the water. N_2_ was simply purged into the water at the rate of 25 ml/s for 40 min. This condition was taken from the work by Butler et al.^45^ as they have reported that more than 60% of the oxygen can be removed at this optimum condition. The glove box was filled with the Ar, with the oxygen level reduced to the ~ 50 ppm. By taking these preventive measures, the oxygen content of (Nd-RE)_2_Fe_14_B was reduced to ~ 0.7%. Figure 4e shows that the Nd peak is absent when the washed (Nd-RE)_2_Fe_14_B was washed in air. This further confirms that washing with the water reduces the crystallinity of the Nd too. It is well-known fact that the addition of the Nd phase (up to a certain limit) enhances the magnetic properties especially, coercivity^12^.

**Figure 5:**
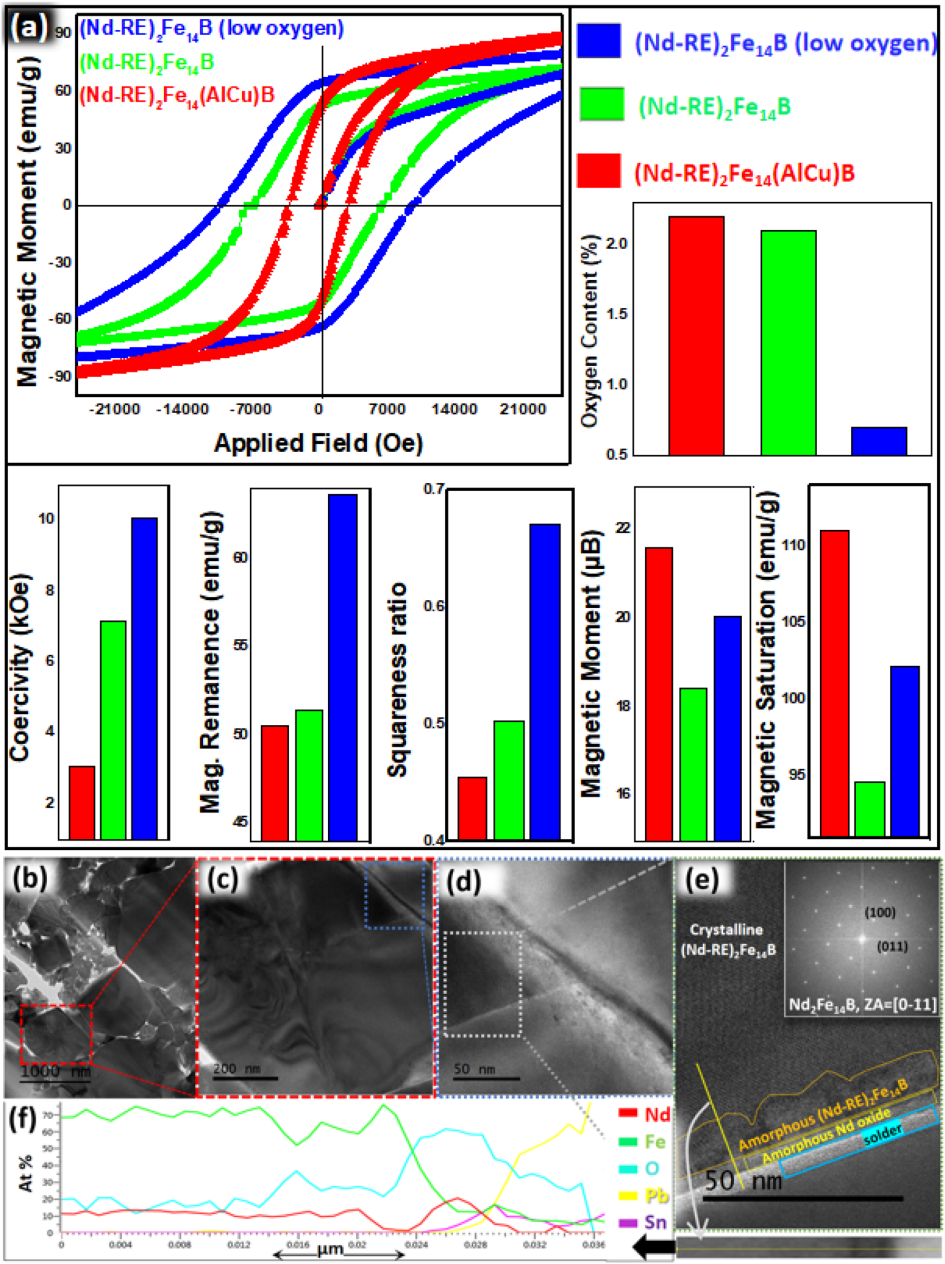
(**a**) Magnetic properties and oxygen content of all products (**b**–**d**) LAADF-STEM image of Nd_2_Fe_14_B (**e**) HAADF-STEM image from (**d**) (**f**) EDS line profile from (**e**).

LAADF-STEM images (Fig. 5b–d) were zoomed in to investigate the micro-structure of the magnetic particles. Line EDS mapping from the HAADF-STEM image was taken and studies to determine the degree of oxidation near the grain boundary (Fig. 5e, f). Pr, Dy, and Tb are substituted inside the (Nd-RE)_2_Fe_14_B, hence their EDS mapping was not studied to avoid the complexity of data. It was found that oxygen content suddenly increased near the grain boundary because these parts of the grain are directly exposed to the water during the washing process. Pb and Sn detected in TEM-EDS come from the solder.

It is evident from the XRD patterns (Fig. 4e) that Cu and Al reduce the crystallinity of the final product, as well as few crystal facets (e.g. [216],[324]), are also missing. The exact mechanism that how Cu and Al affects the (Nd-RE)_2_Fe_14_B crystal is yet unknown but most probably Cu and Al interfere during the diffusion of RE, Fe, and B. Poor crystallization after reduction-diffusion is the major factor that reduced the magnetic properties of (Nd-RE)_2_Fe_14_(AlCu)B. The second reason for the poor magnetic properties of (Nd-RE)_2_Fe_14_(AlCu)B is the non-magnetic behavior of Cu. Cu has an electronic configuration of [Ar] 3d^10^ 4s^1^ which indicates the absence of unpaired electrons. Hence Cu and its alloys tend to be non-magnetic. In the bulk sintered (Nd-RE)_2_Fe_14_B magnets, Cu is used to decouple the large magnetic grains. Nd-Cu reduces the effect of the surge of domain wall removal in bulk magnets and enhances the coercivity. But in the case of magnetic particles, the grain decoupling phenomenon does not work efficiently. Hence non-magnetic Nd-Cu phase reduces the overall magnetic properties of the (Nd-RE)_2_Fe_14_(Cu-Al)B. Al is detectable in the final product but it is in the amorphous form. Effect of amorphous Al or its possible amorphous alloy on the magnetic properties is not clear.

Decreasing order of the Ms value in all the products is given as (Nd-RE)_2_Fe_14_(AlCu)B > (Nd-RE)_2_Fe_14_B-(low oxygen) > (Nd-RE)_2_Fe_14_B. (Nd-RE)_2_Fe_14_(AlCu)B has highest Ms value, which indicates the presence of amorphous Fe or low anisotropy field. (Nd-RE)_2_Fe_14_B-(low oxygen) has slightly higher Ms value as compared to the (Nd-RE)_2_Fe_14_B that refers to the enhanced Mr value of (Nd-RE)_2_Fe_14_B-(low oxygen), which slightly affected the Ms. However, this enhancement in the Ms value is much higher as compared to the Ms value, which is also evident in the squareness ratio graph (Fig. 5a) showing the enhanced squareness ratio of (Nd-RE)_2_Fe_14_B-(low oxygen).

(Nd-RE)_2_Fe_14_(AlCu)B exhibit the highest magnetic moment (21.6 μB) and M_s_ (1.057 T) value among all three products. Higher magnetic moment and low M_r_ value of (Nd-RE)_2_Fe_14_(AlCu)B refer to the low anisotropic field or presence of amorphous soft magnetic phase (e.g. Fe). Higher M_r_ value of (Nd-RE)_2_Fe_14_B-(low oxygen) contributes to its highest squareness ratio among all three products (Fig. 5a). Individual values of magnetic moments of (Nd-RE)_2_Fe_14_(AlCu)B, (Nd-RE)_2_Fe_14_B, and (Nd-RE)_2_Fe_14_B-(low oxygen) were determined as 21.06, 20.5, and 18.4 μB, respectively. These values of magnetic moments were determined by the M_s_ values from hysteresis loops (Fig. 5a). Complete hysteresis loops with applied magnetic field range of − 9.5 to 9.5 Tesla are provided in supporting information as Fig. S12.

Reduction in magnetic moment enhanced the coercivity. From the hysteresis loops, coercivity values of (Nd-RE)_2_Fe_14_(AlCu)B, (Nd-RE)_2_Fe_14_B, and (Nd-RE)_2_Fe_14_B-(low oxygen) were determined as 242.71, 568.13 and 800.55 kA/m, respectively. (Nd-RE)_2_Fe_14_B-(low oxygen) showed the highest coercivity due to low oxygen content, better crystallinity, and least magnetic moment. Oxygen content values of (Nd-RE)_2_Fe_14_(AlCu)B, (Nd-RE)_2_Fe_14_B and (Nd-RE)_2_Fe_14_B-(low oxygen) are recorded as 2.2, 2.1 and 0.7%. The increasing order of coercivity is (Nd-RE)_2_Fe_14_(AlCu)B < (Nd-RE)_2_Fe_14_B < (Nd-RE)_2_Fe_14_B-(low oxygen) and the decreasing order of magnetic moment is (Nd-RE)_2_Fe_14_(AlCu)B > (Nd-RE)_2_Fe_14_B-(low oxygen) > (Nd-RE)_2_Fe_14_B. Individual M_r_ values of (Nd-RE)_2_Fe_14_(AlCu)B, (Nd-RE)_2_Fe_14_B, and (Nd-RE)_2_Fe_14_B-(low oxygen) were determined as 0.481, 0.489, 0.605 T, respectively. M_r_ (emu/g), M_s_ (emu/g), squareness ratio (S_q_), magnetic moment (μB), and coercivity (H_c_), for the all the products are shown in Fig. 5a and Table S2, comparatively.

## Conclusion

Contaminated Nd_2_Fe_14_B sludge was recycled by four-step chemical process. The process consisted of leaching of sludge in H_2_SO_4_, removal of impurities by selective co-precipitation, annealing, and calciothermic reduction diffusion. Al^3+^ and Cu^3+^ were removed by co-precipitation at pH 6 and residual carbon was removed by annealing at 800 °C. CaO byproduct was separated by washing in the glove box, in presence of very low level of oxygen that reduced the oxidation of (Nd-RE)_2_Fe_14_B produced. Removal of impurities and low oxygen content (~ 50 ppm) tripled the coercivity (over 800 kA/m) and enhanced the M_r_ value up to 0.605 T. The Method reported in this study is simple, eco-friendly, and energy efficient.

## Supplementary Information

Supplementary Information.
