# Supplementary material for: Four-step eco-friendly energy efficient recycling of contaminated Nd2Fe14B sludge and coercivity enhancement by reducing oxygen content
Source: Sci Rep. 2021 Nov 15;11:22255. doi: 10.1038/s41598-021-01382-4 (PMC8593190; doi:10.1038/s41598-021-01382-4)
Supplement: Supplementary file 1 — Supplementary Information. [file 41598_2021_1382_MOESM1_ESM.docx]

**Four Step Eco-friendly Energy Efficient Recycling of Contaminated Nd_2_Fe_14_B Sludge and Coercivity Enhancement by Reducing Oxygen Content**

Syed Kamran Haider^a,b,c^, Dongsoo Kim^a,b,*^ , Young Soo Kang^c*^

^a^ Convergence research center for development of mineral resources, Korea Institute of Geoscience and Mineral Resources, Daejeon 34132, South Korea.

^b^ Powder & Ceramics Division, Korea Institute of Materials Science, Changwon, Gyeongnam 51508, South Korea.

^c^ Department of Chemistry, Sogang University, 35, Baekbeomro, Mapogu, Seoul, 04107, South Korea.

*Corresponding author

Email: [yskang@sogang.ac.kr](mailto:yskang@sogang.ac.kr)

Telephone: +82-2-702-6379

1. **Analysis of the Cu and Al hydroxides**

Hydroxides of Cu and Al were separated and dried in the oven at 250 ^o^C for one hour. Because of low temperature drying hydroxides were partially converted to the oxides and still most part of the precipitates looks amorphous (Fig. S-1).SEM and SEM-EDS images of the same powder is given as Fig. S-2.

1. **Optimization of co-precipitation pH**

Co-precipitation method in this work was similar to the process introduced by the *Ma et al.^1^* Both of the them used 3.5 M NaOH for co-precipitation (same as this work) but in their work pH was raised up to 10. In this work during the co-precipitation, pH was raised up to 10,11,12 and 13 in separate experiments was centrifuged at lower rpm (3500rpm/1min). However, it was found the by increasing the pH percentage yield increased significantly (Fig. S-3). Furthermore, after co-precipitation at pH 10, some of the precipitates were suspended in the solution and it took more than 2 hours for hydroxide precipitates to settle down. But after co-precipitation at pH 13, it took ~15 minutes for hydroxide precipitates to settled down. When precipitates settled down completely, the separation of the byproduct (solution of the Na_2_SO_4_ and NaOH dissolved in the water) becomes easy. More than 80% of the byproduct can be removed just by decanting, without any centrifugation. In order to remove the by-products of co-precipitation *Ma et al.^1^.* used centrifugation at 8000 rpm for 5 minutes. In this work after the co-precipitation centrifugation at 3500 rpm for 1 minute removed all the byproducts.

1. **Proposed experimental process for HDDR, magnetization and resin binding:**

(Nd-RE)_2_Fe_14_B powder synthesized by the recycling of the undergoes the Hydrogen decrepitation deabsorbation recombination (HDDR). Processed magnetic powder is magnetized and changed to the bulk magnet by resin binding. Details of the proposed HDDR process are given below.

**3.1 HDDR treatment and magnetization:**

20 g was of the ( Nd-RE)_2_Fe_14_B powder will be loaded into a stainless steel tube, and then will be placed inside an Inconel-tube furnace. The system will be evacuated using a rotary pump (to ~10^-2^ mbar) and the Nd_2_Fe_14_B powder will be hydrogen decrepitated using a pressure of 2 bar of hydrogen at room temperature to produce a hydrided powder. The system will be subsequently evacuated and heated under vacuum up to 920 ^o^C. Meanwhile, hydrogen desorption trace will be measured by using a Lesker vacuum gauge with attached data logger to monitor the pressure in the furnace tube and the temperature of the powder. On approaching the desired temperature hydrogen will be introduced at a rate 16 mbar/m into the processing pressure of 1500 mbar and then will be held for 30 min, giving an overall processing time of roughly 2 h. The hydrogen pressure will be reduced at a rate of 100 mbar/min until the pressure will approach same as the rotary pump vacuum. Then powder will be cooled quickly by rolling the furnace of the tube and placing a water-cooled copper coil over the tube. In this way, cooling of the powder up to 500 ^o^C will occur in less than 5 min, by preventing excessive grain growth at elevated temperature. Once cooled, the samples will be removed from the furnace, lightly ground using a pestle and mortar to break up any agglomerated powder and then prepared for testing. Powder samples of 100 mg will be set in wax in cylindrical sample holders and will be aligned using a constant magnetic field of 1.7 T by an electromagnet in order to align the particles in the preferential c-axis direction. Finally, the samples will be pulse magnetized in the c-axis direction, using a field of 4 T from a capacitor discharge pulse magnetizer.

**3.2 Synthesis of the resin bonded magnets:**

After the magnetization, magnetic particles will be ready for the resin binding process. A review of the resin binding process is given below. In order to prepare the bonded magnets, the compression, injection molding, extrusion and calendaring processes are used. For injection molding and extrusion process LDPE powder (45 μm) is the ideal choice. However, for the calendering machine nitrile rubber is good option. For calendering and injection molding 70 volume percent Nd_2_Fe_14_B should be used with the resin. However, for extrusion process 75 volume percent Nd_2_Fe_14_B is more appropriate. Calendaring can produce 0.25 inch magnet sheet, meanwhile, small cylindrical magnets can be produced by the injection molding and extrusion. To obtain the good results in the resin bonding process, following points needed to be addressed.

1- Because of using of inappropriate equipment (e.g iron extruder) magnetic particles could be wasted/in-homogeneously mixed.

2- It is found that Nd_2_Fe_14_B powder produced by reduction diffusion contains ~2% oxygen (wt%). Meanwhile, commercial Nd_2_Fe_14_B powder contained 0.4% oxygen. We assume that important reason for the lower oxygen content in the commercial Nd_2_Fe_14_B powder is their particle size. Average particle size of the commercial powder is ~25 μm. MPC or resin bonding at higher temperature raised the oxygen content further, that lead to the poor magnetic properties. Oxygen content of Nd_2_Fe_14_B produced by reduction-diffusion method can be reduced by washing the Nd_2_Fe_14_B particles in the glove box.

**4. Effect of Ho, Zr, Ga, Ni and Co impurities on the magnetic properties:**

In order to study the effect of Ho, Zr, Ga, Ni and Co impurities, a separate experiment was performed. In this experiment composition of the precursors was kept same as sludge but Ho, Zr, Ga, Ni and Co were not added. All other experimental parameters were kept same. As shown in the hysterises loop below, detectable difference (Figure S-13) was observed in the magnetic properties of the products of both the experiments.

A comparison of the magnetic properties prepared by both the experiments is shown in the table 1. Reduced Mr value (~ 8%) and enhanced coercivity (~ 4%) in the (Nd-RE)_2_Fe_14_B particles prepared from the sludge may refer to the presence of the non-magnetic impurity/impurities.

**Figures and figure captions:**


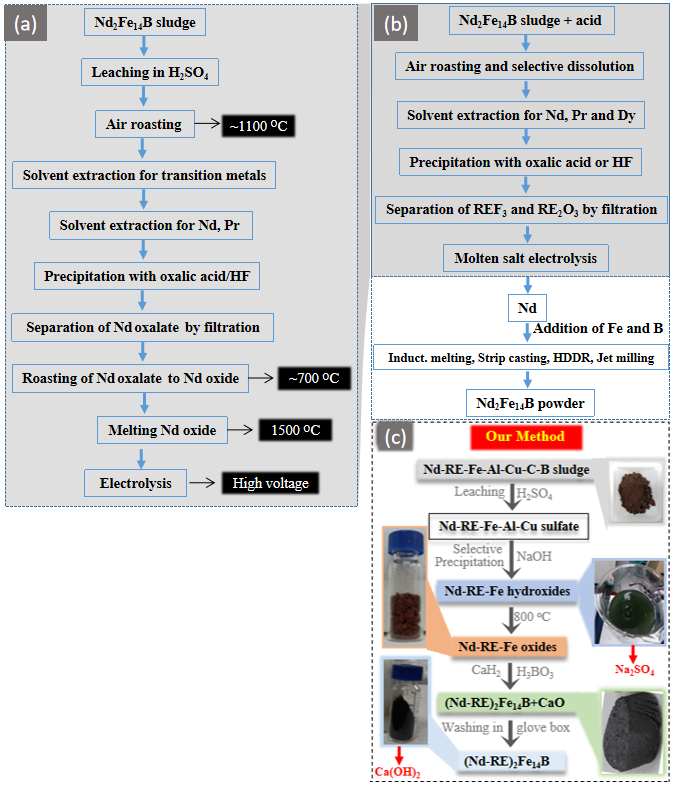


Fig. S-1 (a) Partial expanded part of the Figure 1-b (b) Common physico-chemical method for the recycling of Nd_2_Fe_14_B sludge used these days (c) Our experimental process.


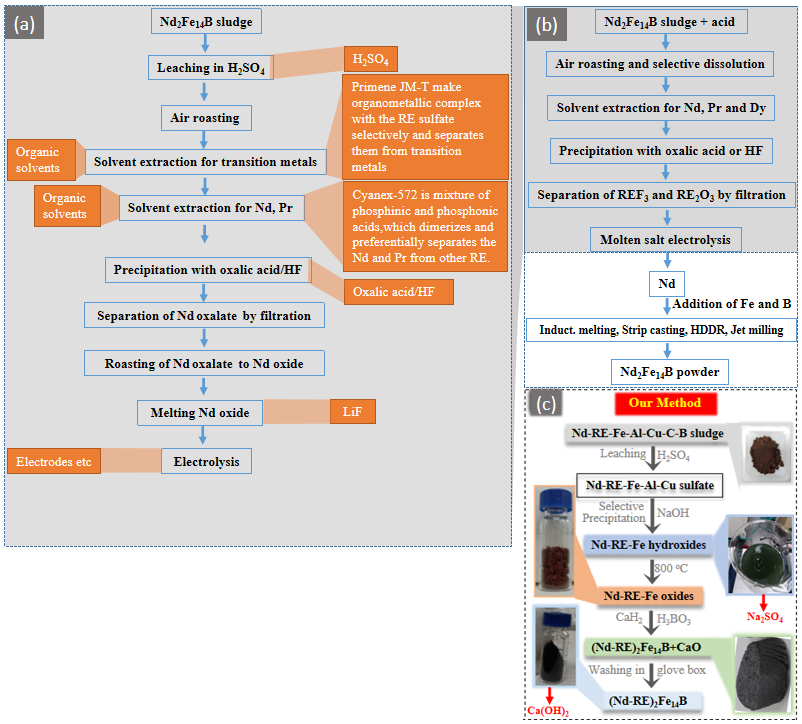


Fig. S-2 (a) Partially expanded part of the Figure 1-b (b) Common physico-chemical method for the recycling of Nd_2_Fe_14_B sludge used these days (c) Our experimental process.


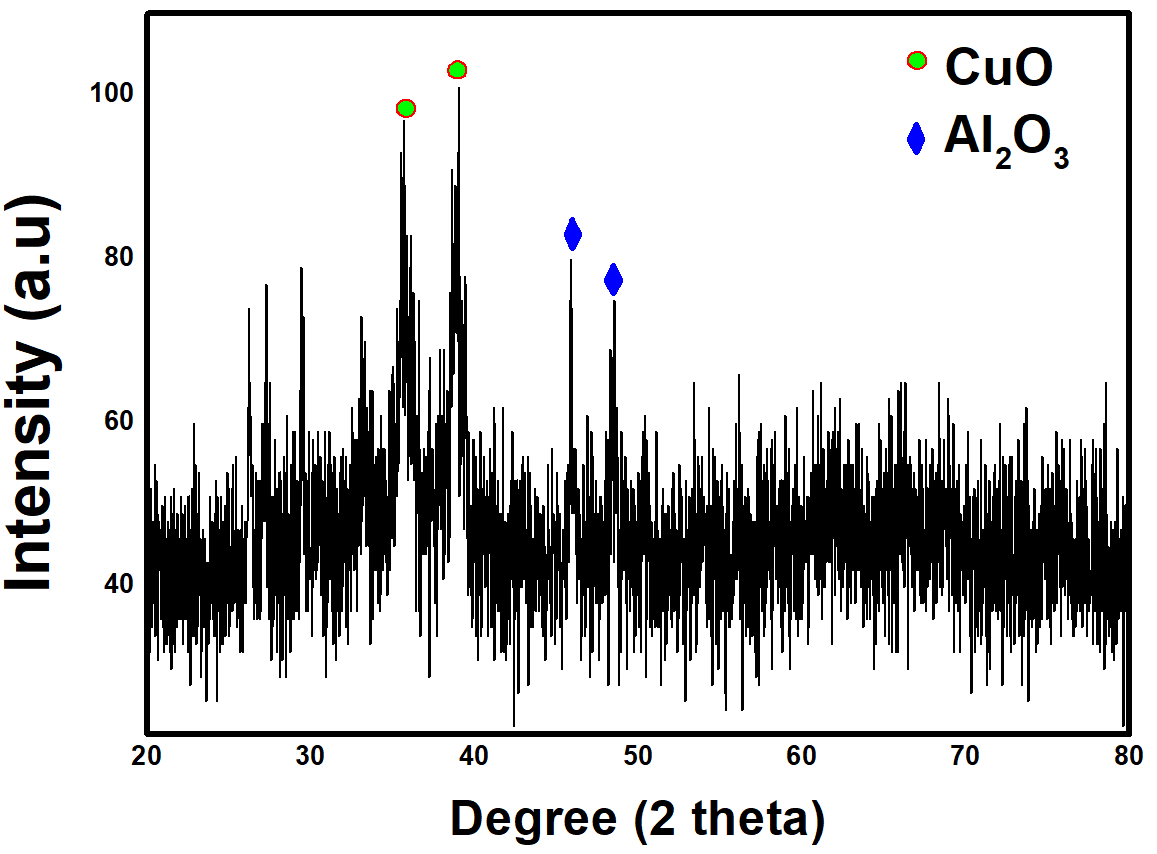


Fig. S-3. XRD patterns of the dried (at 250 ^o^C) hydroxide precipitates of Cu and Al.


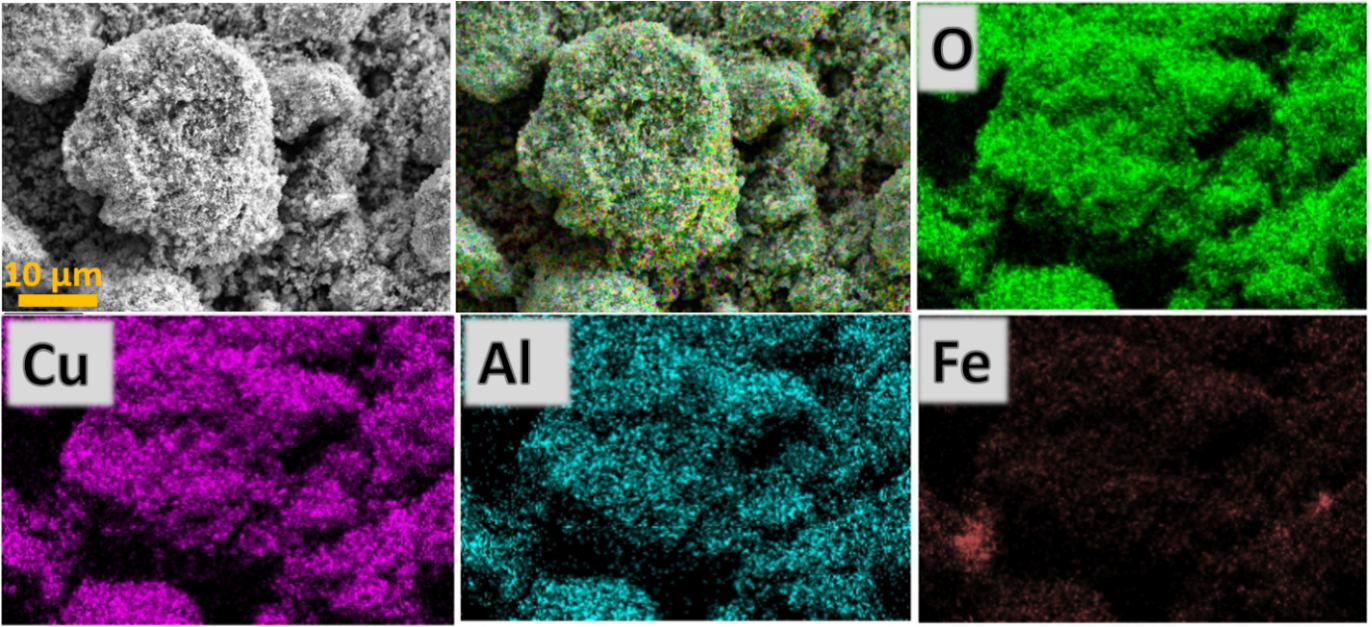


Fig. S-4. SEM and SEM-EDS images of the dried (at 250 ^o^C) hydroxide precipitates of Cu and Al.


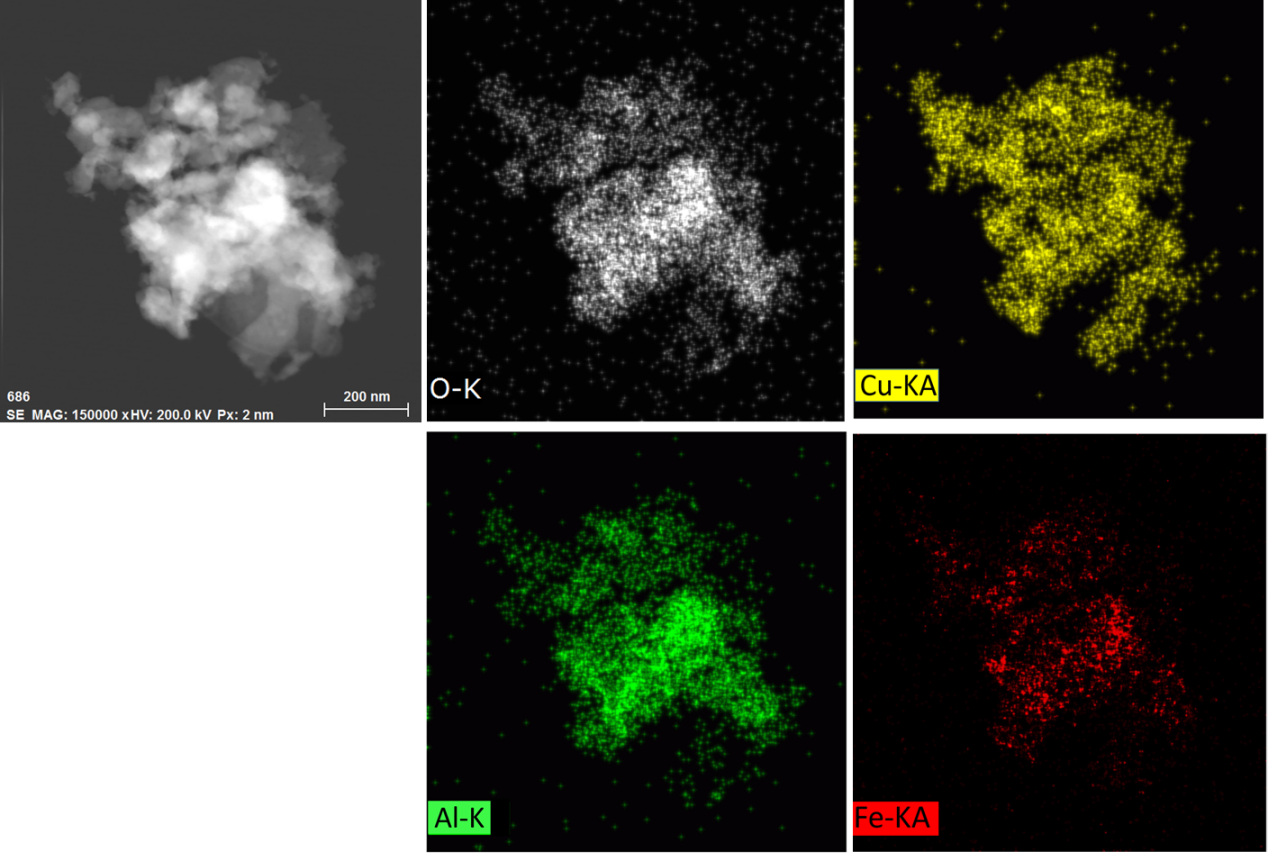


Fig. S-5. TEM and SEM-EDS images of the dried hydroxide precipitates, precipitated at pH 6.

Fig. S-6. Percentage yield of the hydroxide precipitates produced at different pH.


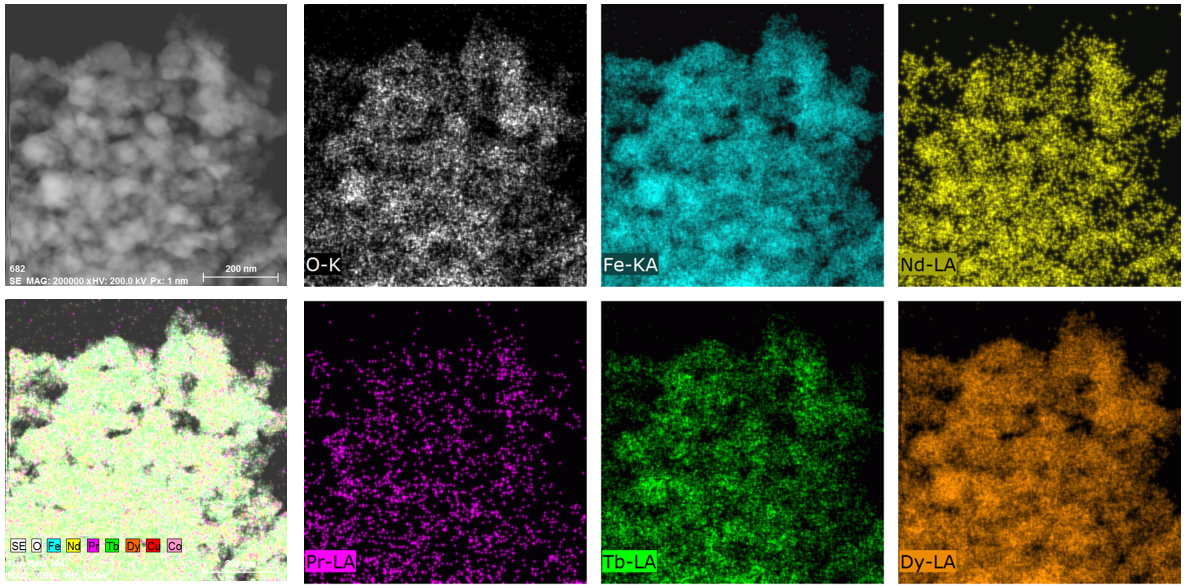


Fig. S-7. TEM-EDS images of Nd-RE-Fe hydroxide precipitates.


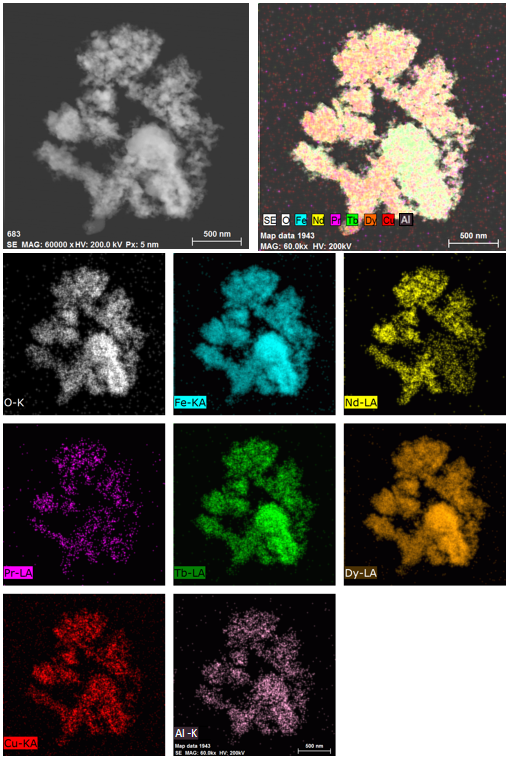


Fig. S-8. TEM-EDS images of Nd-RE-Fe-Cu-Al- hydroxide precipitates


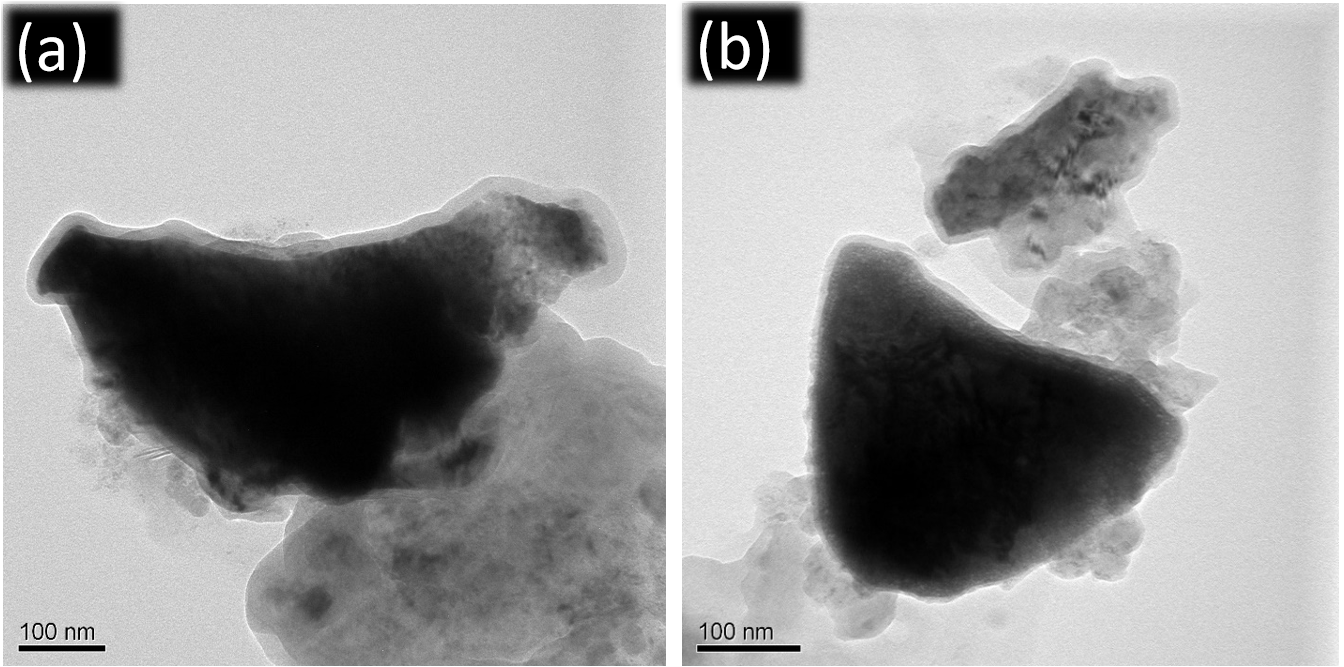


Fig. S-9.TEM images of (a) (Nd-RE)_2_Fe_14_B and (b) (Nd-RE)_2_Fe_141_B (low oxygen)


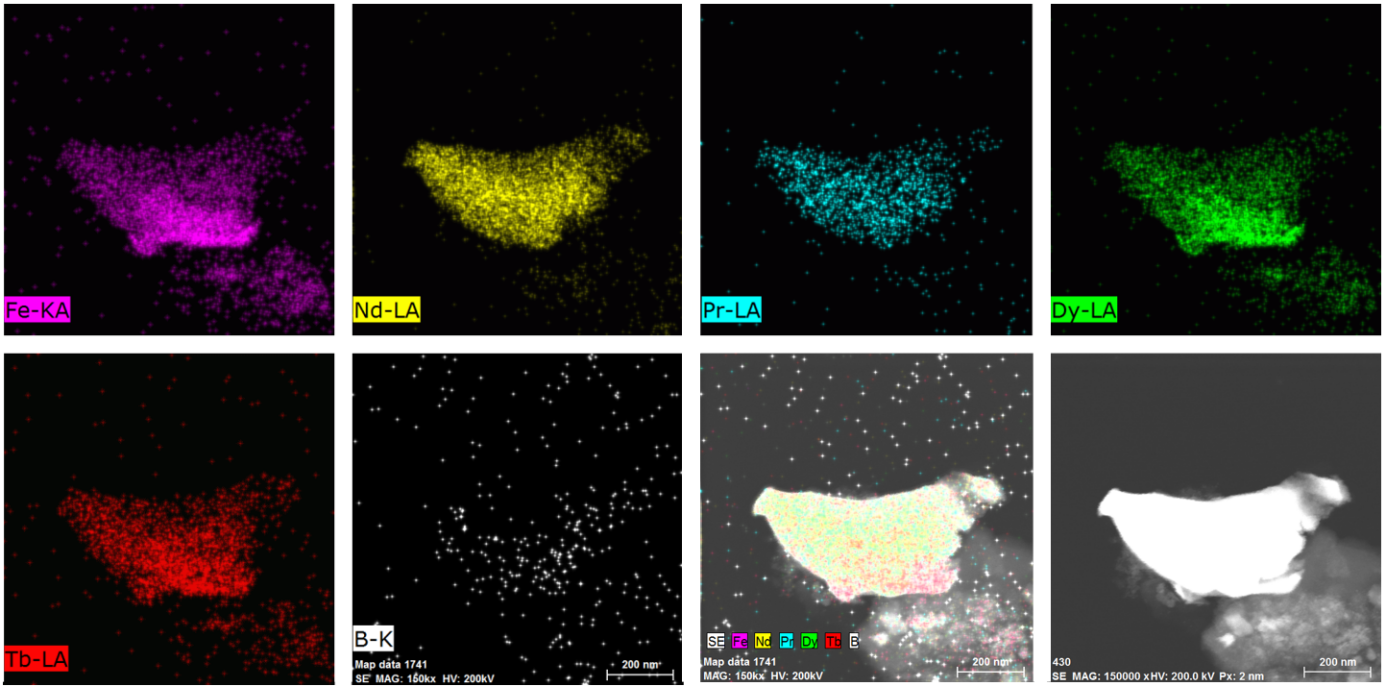


Fig. S-10. TEM-EDS images of (Nd-RE)_2_Fe_14_B.


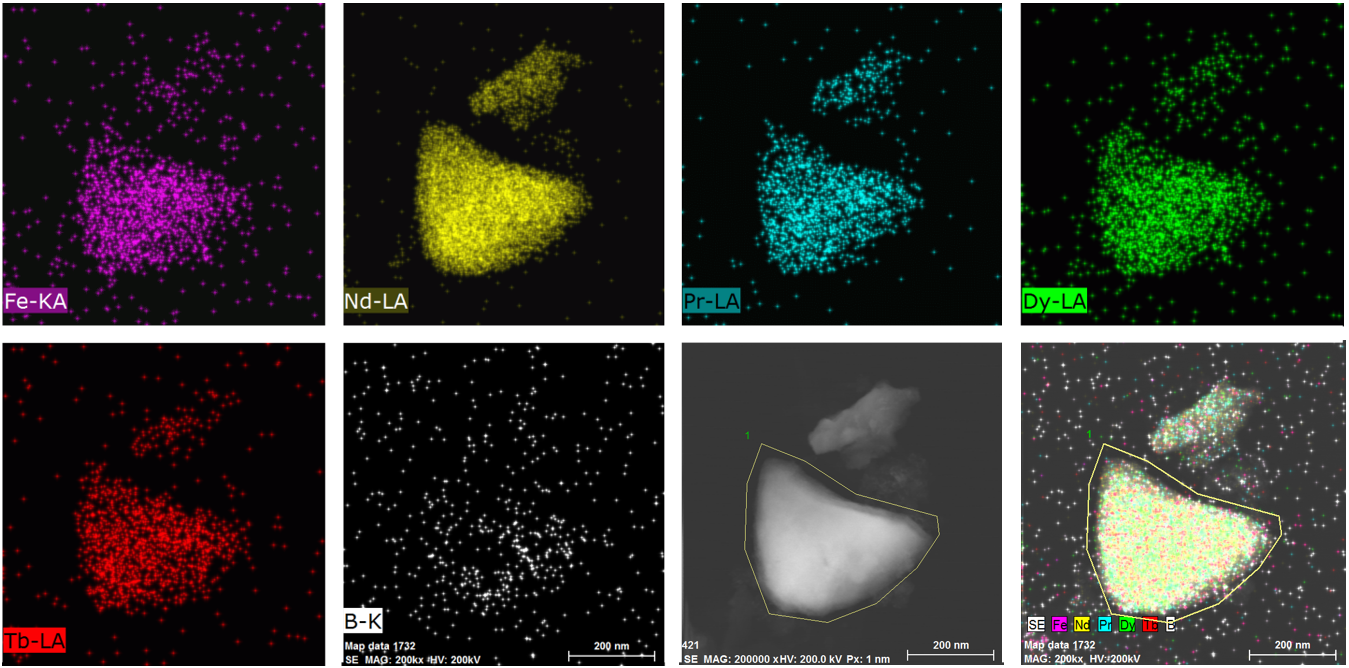


Fig. S-11. TEM-EDS images of (Nd-RE)_2_Fe_14_B (low oxygen).

Fig. S-12. Magnetic hysteresis loop of (Nd-RE)_2_Fe_14_(CuAl)B, (Nd-RE)_2_Fe_14_B and (Nd-RE)_2_Fe_14_B (low oxygen).


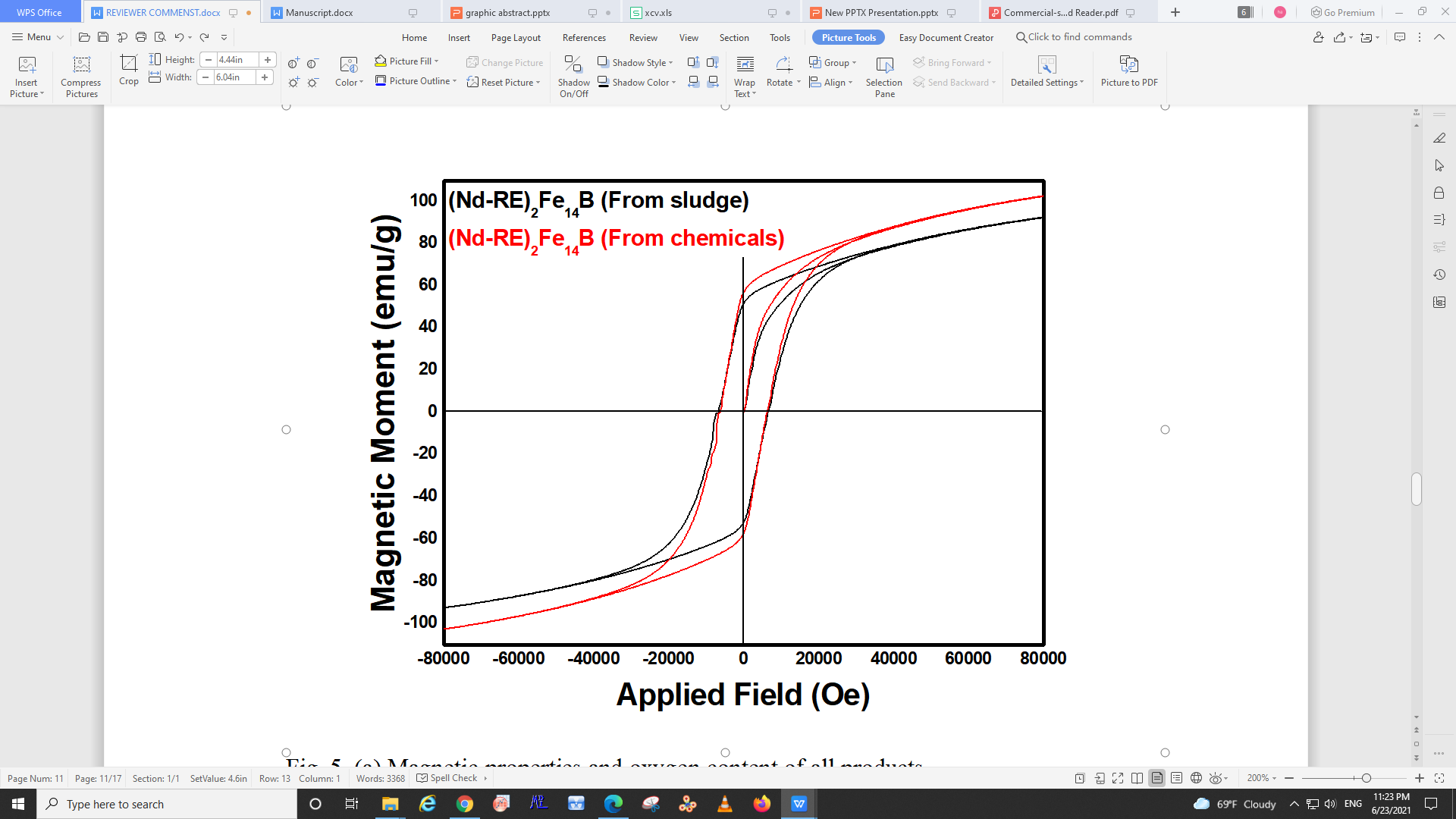


Fig. S-13. Hysteresis loop of (Nd-RE)_2_Fe_14_B, prepared from Chemicals and sludge.

| **Source** | **Hc (kOe)** | **Mr (emu/g)** |
| --- | --- | --- |
| (Nd-RE)_2_Fe_14_B from Chemicals | 6.31 | 55.66 |
| (Nd-RE)_2_Fe_14_B from sludge | 6.62 | 51.52 |

Table. S-1. Comparison of the magnetic properties of (Nd-RE)_2_Fe_14_B, prepared from Chemicals and sludge.

| **Product** | **Coercivity (kA/m)** | **Mr (T)** | **Ms (T)** | **Squareness ratio** | **Mag. Mom. (μB)** |
| --- | --- | --- | --- | --- | --- |
| (Nd-RE)_2_Fe_14_(AlCu)B | 242.71 | 0.481 | 1.057 | 0.45 | 21.6 |
| (Nd-RE)_2_Fe_14_B | 568.13 | 0.489 | 0.973 | 0.50 | 20.05 |
| (Nd-RE)_2_Fe_14_B(low oxy) | 800.55 | 0.605 | 0.902 | 0.67 | 18.43 |

Table S-2. Magnetic properties of (Nd-RE)_2_Fe_14_(AlCu)B, (Nd-RE)_2_Fe_14_B, (Nd-RE)_2_Fe_14_B (low oxygen).

**References**

# Ma, Z. H., Zhang, T. L. & Jiang, C. B. A facile synthesis of high performance SmCo_5_ nanoparticles. *Chem. Eng. J.* 264, 610– 616 (2015). [https://doi.org/10.1016/j.cej.2014.11.138](https://doi.org/10.1016/j.cej.2014.11.138)
